# Optimization of Lipopeptide Biosurfactant Production by *Salibacterium* sp. 4CTb in Batch Stirred-Tank Bioreactors

**DOI:** 10.3390/microorganisms10050983

**Published:** 2022-05-08

**Authors:** José Martín Márquez-Villa, Juan Carlos Mateos-Díaz, Jorge Alberto Rodríguez-González, Rosa María Camacho-Ruíz

**Affiliations:** Department of Industrial Biotechnology, CIATEJ-CONACyT, Zapopan 45019, Mexico; jmmv.ipn@gmail.com (J.M.M.-V.); jcmateos@ciatej.mx (J.C.M.-D.); jrodriguez@ciatej.mx (J.A.R.-G.)

**Keywords:** extremophiles, microbial processes, biosurfactant, bioreactor, DOE

## Abstract

Halophilic microorganisms are potentially capable as platforms to produce low-cost biosurfactants. However, the robustness of bioprocesses is still a challenge and, therefore, it is essential to understand the effects of microbiological culture conditions through bioreactor engineering. Based on a design of experiments (DOE) and a response surface methodology (RSM) tailored and taken from the literature, the present work focuses on the evaluation of a composite central design (CCD) under batch cultures in stirred-tank bioreactors with the halophilic bacteria *Salibacterium* sp. 4CTb in order to determine the operative conditions that favor mass transfer and optimize the production of a lipopeptide. The results obtained showed profiles highlighting the most favorable culture conditions, which lead to an emulsification index (E_24_%) higher than 70%. Moreover, through the behavior of dissolved oxygen (DO), it was possible to experimentally evaluate the higher volumetric coefficient of mass transfer in the presence of lipopeptide (*k_L_a* = 31 1/h) as a key criterion for the synthesis of the biosurfactant on further cell expansion.

## 1. Introduction

Extremophilic microorganisms, it turns out, are life forms that thrive and perform all their metabolic activity in locations that would be harmful to other life forms from an anthropocentric point of view [1]. In particular, halophilic microorganisms are described to possess tolerance to hypersalinity up to 30% *w*/*v* NaCl in their intracellular content and are geographically abundant in extreme environments, such as the Dead Sea, Antarctic lakes, and Cuatro Cienegas (Mexico), among others [2,3,4,5]. The vast number of halophilic microorganisms have versatile biotechnological potentials due to their intrinsic resistance to developing metabolic function under conditions of low water availability and high salinity [6]. In addition, they are capable of producing extracellular and intracellular enzymes and surface-active agents [7,8].

Surface-active agents, known as surfactants, are a broad class of amphiphilic compounds that are widely used in hydrocarbon remediation, cleaners, pesticides, and health products, among others [9,10,11]. Nonetheless, chemical surfactants outperform the market distribution due to the reduced cost of production at high volumes, even considering the damage they represent to the environment. Diverse industries are conducting research in order to find biocompatible sources of biological surface-active agents (SABs) [12,13,14,15]. Similar to their chemical counterparts, biosurfactants are amphiphilic molecules with comparable physicochemical properties [16]. Based on their structure and microbial origin, biosurfactants can be divided into two broad categories: low molecular weight compounds and high molecular weight compounds [17,18,19]. The low molecular weight class includes biosurfactants, especially glycolipids and lipopeptides; the high molecular weight class includes amphipathic polysaccharides, lipopolysaccharides, and lipoproteins [20,21,22]. In the late 1960s [23], the main use of biosurfactants was as dispersants in oil spill contaminated sites.

Microbial biosurfactants are commonly known to belong to the class of secondary metabolites produced in the stationary phase of growth [20,24]. Although, some studies [7] have provided evidence that biosurfactants could be synthesized during the exponential phase as primary metabolites. Biosurfactants exhibit diverse environmental potentials, such as production from renewable sources, biodegradability, surface and interface activity, and antibiotic and antiviral activity [23,25,26,27,28,29,30]. In particular, the environmental biodegradability potential, which characterizes biosurfactants, is attributed to their structural components, such as carbohydrates, amino acids and proteins, and lipids [31]. Therefore, they do not accumulate in environmental matrices to access the various natural degradation processes.

Even if biosurfactants are not yet economically comparable to chemical surfactants to date, the global biosurfactants market size during 2020 was over USD 1.75 billion and based on this increase, it is expected to grow at a CAGR of over 5.5% between 2021 and 2027, associated with the changing consumer perception towards the environmental impact generated by synthetic surfactants [32].

Challenging approaches have been described to exploit halophiles at the bioreactor scale, such as the high substrate cost, oxidation in bioreactor structures and sensors, narrow titers in native strains, and foaming, among others [1,15,25]. However, advances have been made through engineering and statistical techniques in the field of biotechnology, to overcome barriers, such as single-use bioreactor design [33], and enhance the mass transfer rates (agitation, aeration, pH, temperature, and culture medium) [34,35]; under the remarkable statistical techniques of Box–Wilson, Box–Behnken, and Placket–Burman, among others [36], to reach optimal conditions. Moreover, molecular biology has been a breakthrough strategy for halophiles by cloning the genes encoding a specific product into a heterologous expression system, making it a tailor-made technology [37,38,39].

*Salibacterium* sp. 4CTb has been shown in previous research to be a polyextremophilic microorganism, and one of interest for its potential to produce the smallest lipopeptide reported to date [7]. For reasons of clarity, this research aims to provide insight into microorganisms that are capable of growing under harsh conditions and exploit their biomolecules through scale-up production platforms, such as bioreactors, and channel them towards the development of innovative technologies in oil recovery, bioremediation, and versatile tools for bioprocess operations with greater robustness.

## 2. Materials and Methods

### 2.1. Halophilic Bacteria Strain

The bacterial strain was isolated from a saline soil sample from Cuatro Cienegas Valley, Coahuila, Mexico [40]. The identification was performed by conducting molecular sequencing analysis through the 16S rRNA gene, as described by Barbachano-Torres et al. [7]. The obtained 16S rRNA gene sequences were deposited in the NCBI archive under bioproject MG869652.

### 2.2. Media Preparation

The bacteria *Salibacterium* sp. 4CTb was cultivated in an ATCC2185 liquid medium; with the final composition per liter being: 120 g of NaCl, 3 g of glucose, 20 g of MgSO_4_∙7H_2_O, 6 g of yeast extract, 2 g of KCl, 5 g of tryptone, and 0.1 mL of mineral solution. Glucose was employed as a sole source of organic carbon in the media. The mineral solution was composed of 1.32 g of ZnSO_4_∙H_2_O, 0.34 g of MgSO_4_∙H_2_O, 0.82 g of Fe (NH_4_) SO_4_∙6H_2_O, and 0.14 g of CuSO_4_∙5H_2_O. The pH of the medium was adjusted to 9 with NaOH before sterilization. The mineral solution was added after sterilization of the macroelements to avoid precipitation of the components. For solid cultures, bacteriological agar was added at a concentration of 50 g per liter.

### 2.3. Flask Scale Inoculum Cultivation

Reactivation of wild-type strain 4CTb was commenced by thawing a cryovial, in which 10 μL of the sample was plated onto an ATCC2185 solid medium and streaked. Incubation conditions were 37 °C for 3 days until colonies appeared. After colony development, one colony was transferred to a 125 mL Erlenmeyer flask with a working volume of 20 mL of ATCC2185 liquid medium. Incubation conditions were 37 °C and 300 rpm for 3 days [7].

### 2.4. Cell Expansion in Bioreactors

Culture preparations in cell expansion from flask scale to bioreactor were performed until a cell concentration of 1 × $10^{6}$ (cell/mL) was reached. Cell expansion was done in batch cultures, where they were grown in stirred-tank bioreactors. The bioreaction platforms were miniBio250s (Applikon Biotechnology, Delft, The Netherlands) instrumented with pH and DO (Applisens, Delft, The Netherlands) sensors, an L-type open pipe sparger, two Rushton 6-blade impellers, and a my-Control bioconsole for the regulation of the culture parameters. The operating conditions maintained the thermal and chemical scaling criteria between the flask and bioreactor scale, where the temperature of each bioreactor was 37 °C, pH 9, and using the same composition of ATCC2185 liquid medium. The mechanical parameters of the gas supply and agitation were adjusted according to a design of experiments (DOE) consisting of a 2*^k^* factorial optimization design by a central composite design (CCD). Noteworthy, aeration conditions are expressed in terms of one volume of air per volume of medium per minute (vvm).

### 2.5. Determination of Biomass Development

Cell development was monitored by optical density (OD). The culture sample was diluted at 1:20 with a fresh and sterile ATCC2185 medium. Next, the sample was dispensed into a 96-well microplate, where software was configured to detect absorbances at a wavelength of 620 nm using a UV-vis spectrophotometer. All samples were measured in triplicate and analyzed by means and standard deviations.

### 2.6. Determination of Lipopeptide Production

Lipopeptide production was determined by the emulsification index (E_24_%) [41,42], where 1 mL of previously centrifuged culture supernatant (6547 g-forces, 5 min) and 1 mL of hexane were placed in a 5 mL test tube. After adding the components, it was allowed to rest for 24 h and shaken vigorously for 1 min. The emulsification index was determined by Equation (1) based on the relative heights of the system.

(1) $$E_{24}\%=\left(\frac{h_{e}}{h_{t}}\right)*\left(100\right)$$

where $h_{e}$ denotes the emulsion height and $h_{t}$ represents the height of the solution. The heights were measured in centimeters. All samples were measured in triplicate and analyzed by means and standard deviations.

### 2.7. k_L_a Determination

The volumetric mass transfer coefficient (*k_L_a*) was obtained in all bioreactor runs, where the dynamic method was used for the experimental determination of *k_L_a* [43,44,45,46]. Dissolved oxygen (DO) concentration parameters were measured continuously using a polarographic O_2_ sensor (Applisens) and monitored by Lucullus PIMS software (Applikon Biotechnology). The measurement of the respiratory activity of *Salibacterium* sp. 4CTb was performed during its exponential phase, where the gaseous supply was briefly interrupted until the minimum DO value was reached, and then the gas flow was restored until the DO concentration was stable. The mass balance for DO can be expressed as:

(2) $$\frac{dC}{dt}=OTR-OUR$$

where *dC*/*dt* represents the rate of oxygen accumulation in the liquid phase, *OTR* represents the rate of oxygen transfer from the gas to the liquid, and *OUR* is the rate of oxygen uptake by the microorganism. Therefore, the expression of the experimental values of *k_L_a* in linear terms is represented by Equation (3):

(3) $$\left(k_{L}a\right)*\left(t_{2}-t_{1}\right)=Ln\left(\frac{C_{l,\infty }-C_{l,t1}}{C_{l,\infty }-C_{l,t2}}\right)$$

where *k_L_a* is the volumetric oxygen transfer coefficient, *t* is the time variation, $C_{l,\infty }$ is the oxygen saturation concentration, and $C_{l,t}$ is the oxygen concentration at a given time. No experiments without microbiological culture were performed.

### 2.8. Statistical Analysis

#### Central Composite Design for Optimization

The optimization process was designed through the construction of a second-order model with central composite designs (CCD) [47,48]. The full 2*^k^* factorial design type model for lipopeptide optimization was designed based on the agitation and aeration factors, where *k* is the number of factors. However, the biomass data were also fitted to the model. In addition, the center point has the function of replicating the experiments to eliminate variance, experimental error, or improve the precision; therefore, three replications of the center point were run. Moreover, the four axial points were used to generate the response surfaces. Based on the above, it was proposed to determine the effect of aeration and agitation on lipopeptide production. The $\mu_{y}$ was related to the response variable by employing the full quadratic model expressed as follows in Equation (4):

(4) $$\mu_{y}=\beta_{0}+\overset{k}{\underset{i=1}{\sum }}\left(\beta_{i}\right)*\left(X_{i}\right)+\overset{k}{\underset{i=1}{\sum }}(\beta_{ii})*\left(X_{i}^{2}\right)+\overset{k}{\underset{j=2}{\sum }}\overset{j-1}{\underset{i=1}{\sum }}(\beta_{i,j})\;*\left(X_{i}\right)*\left(X_{j}\right)+\epsilon$$

where $\beta_{0}$ was the intercept coefficient, $X_{i}$ and $X_{j}$ were coded independent variables, $\beta_{i}$ and $\beta_{ii}$ were the coefficients corresponding to the linear and quadratic coefficients, respectively, and $\beta_{i,j}$ was the coefficient of the interaction products. In the model, the coefficient correspondent to error (ϵ) was neglected due to its low significance. Table 1 shows how the coded variables are manipulated mathematically and the real variables are those characterized in the process. The real variables (Z) describe the levels, where Z1 explores the aeration profiles and Z2 the stirring profiles. The axial points were generated by four additional experiments under an α = 1.41, which is a parameter in the CCD that can provide the property of rotation. This property was achieved by $\alpha ={\left(2^{k}\right)}^{\frac{1}{4}}$.

## 3. Results and Discussion

### 3.1. Performance of Salibacterium sp. 4CTb on Biomass and E_24_%

Among the key parameters, where mass transfer rates are improved to yield the lipopeptide biosurfactant production, it has been described that a critical parameter is the nitrogen composition of the culture medium [30,34,49]. Therefore, in previous studies [7] the ATCC2185 culture medium was enriched with yeast extract to an optimal concentration (6 g/L) based on the highest emulsification index that could be achieved to potentially supplement the cultures described in this work. However, the nitrogen determination tests, which provide evidence for the culture stage where the nitrogen was completely depleted, were not performed.

Regardless, when *Salibacterium* sp. 4CTb and other *Bacillus* species are enriched with yeast extract, biosurfactant production is enhanced by the presence of L-Leucine and L-Arginine as sources of nitrogen and direct precursors for SABs synthesis [50]. Considering that the chemical composition was no longer an issue to improve the lipopeptide production by *Salibacterium* sp. 4CTb, the optimization of mechanical parameters for the bioreactor scale is described below.

The profiles explored for each culture were performed based on duplicate experiments to guarantee the repeatability of the runs, where the influence of the aeration and agitation parameters were evaluated in various design combinations of experiments to determine the optimal emulsification index (E_24_%) and how biomass development is involved. This is due to barely any research conducted in lipopeptide biosurfactant production by *Salibacterium* sp. 4CTb through stirred-tank bioreactors.

**Figure 1:**
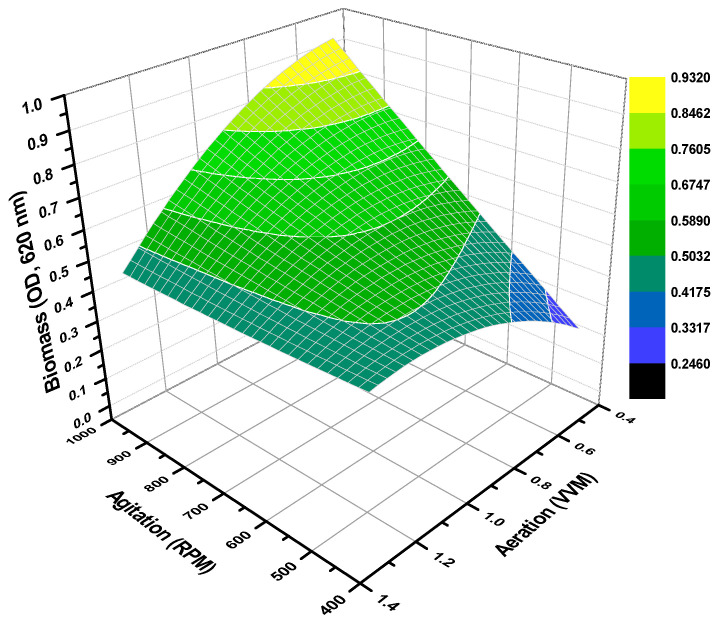
Theoretical biomass values calculated by the quadratic model in response surface methodology from experimental values of biomass production.

Figure 1 shows the behavior of biomass and, by analyzing the response surface graph, shows that high values of biomass were reached in higher agitation (rpm) rate profiles and that they were near aeration rates of 0.5 vvm. Clearly, mass transfer mechanisms favor the metabolism of *Salibacterium* sp. 4CTb towards higher biomass production [43].

For instance, the second-order model from CCD describes several response variables as a function of agitation (rpm) and aeration (vvm), such as biomass and the emulsification index (E_24_%). The model for biomass ($\mu_{Biomass}$) is presented as follows in Equation (5); standard errors in parenthesis:

(5) $$\begin{array}{ll} \mu_{Biomass} & =0.6076\left(\pm 0.0430\right)-0.0478\left(\pm 0.0263\right)*X_{1}+0.1165\left(\pm 0.0263\right)*X_{2}\\ & \;-0.0471\left(\pm 0.0314\right)*X_{1}^{2}+0.0034\left(\pm 0.0314\right)*X_{2}^{2}\\ & \;-0.0909\left(\pm 0.0372\right)*X_{1}*X_{2} \end{array}$$

In this case, the model for biomass shows a relative deficiency in the significance of the fit by exhibiting an R^2^ = 0.8623 (P = 0.0327). Based on the model described in Equation (5) and the response surface graph (Figure 1), it is possible to identify the optimal region which favors biomass development in theoretical terms, which is located at the operating conditions of 980 rpm and 0.42 vvm, achieving optical densities of 0.9305. Throughout the experiments, it was observed that due to the high turbulence of the Rushton type impellers and aeration rate profiles—higher than rates of 900 rpm and 0.9 vvm—the drawback of foaming associated with an emulsion formation by lipopeptide was present. In this context, it is well known that foam causes serious operational problems in biomass and biosurfactant production batches, where it is also associated with the presence of extracellular proteins. Solutions have been considered based on the above, such as mechanical devices adapted to the impeller shaft to break the foam generation and the addition of defoaming compounds. Nonetheless, the mechanical breaker has not been practical and the addition of antifoam agents would compromise the quality of the biosurfactant produced [51,52]. Even so, the behavior of biomass development is interesting, but not core to this work.

On the other hand, the behavior of *Salibacterium* sp. 4CTb to produce the lipopeptide, was differently influenced by the operating parameters than the ones observed in biomass production. The first noticeable evidence was the synthesis of the lipopeptide by *Salibacterium* sp. 4CTb during the exponential phase of cell development, where this type of biomolecule has been described [53] to be associated with the secondary metabolism of microorganisms—and this lipopeptide is among the ones of primary metabolism. For instance, the model for the emulsification index (E_24_%) ($\mu_{E_{24}\%}$) is presented as follows in Equation (6); standard errors in parenthesis:

(6) $$\begin{array}{ll} \mu_{E_{24}\%}= & 62.3018\left(\pm 2.0452\right)-0.6724\left(\pm 1.2543\right)*X_{1}-5.8600\left(\pm 1.2543\right)*X_{2}\\ & \;+2.3749\left(\pm 1.4967\right)*X_{1}^{2}-11.4988\left(\pm 1.4967\right)*X_{2}^{2}\\ & \;+8.3281\left(\pm 1.7712\right)*X_{1}*X_{2} \end{array}$$

**Figure 2:**
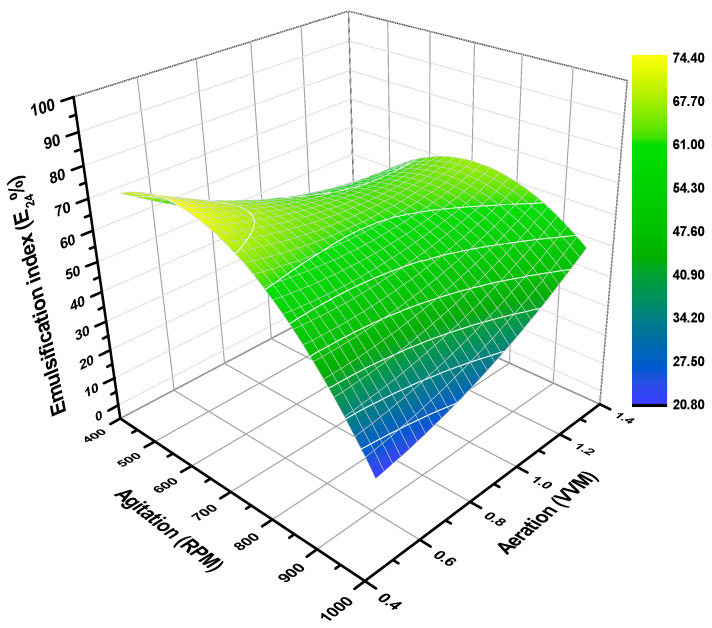
Theoretical values of the emulsification index (E_24_%) were calculated by the quadratic model in response to surface methodology from experimental values of lipopeptide production.

The model for E_24_% presented a significant fit of data, where, from the total variability, it is shown that with R^2^ = 0.9597 (P = 0.0016) the data is explained by the quadratic model on the CCD. Noteworthy, in Figure 2, in the aeration and agitation parameters, it can be noted that at relatively low values, according to the DOE, *Salibacterium* sp. 4CTb readapts its metabolic mechanisms to increase the synthesis of the lipopeptide until reaching an E_24_% higher than 70%. In theoretical terms, and through the response surface graph (Figure 2), an optimal region was located at the operating conditions of 540 rpm and 0.48 vvm, which displayed an E_24_% of 74.55%. Clearly, this behavior can be attributed to the respiratory capacity of *Salibacterium* sp. 4CTb and, that at low concentrations of dissolved oxygen in the medium, it directs energy to produce a biomolecule that enhances the solubilization of oxygen. This result is in agreement with what is reported in the literature [30,34,49,54], where studies employing mesophilic microorganisms, such as *Bacillus subtilis*, *Aureobasidium pullulans*, and *Candida lipolytica*, cultured at a stirred-tank bioreactor scale, show that low aeration or agitation rates at the approximate ranges of 0.38–0.63 vvm and 150–300 rpm tend to perform better in the production of biosurfactants, such as surfactin and rhamnolipid. Likewise, operational difficulties have been addressed due to the high foam generated in the batches and this leads to the novel design of bioreactors or foam collectors as an accessory to the bioreactor. However, the last strategy could increase the detriment of the product due to a loss in the walls and additional handling. An additional criterion that determines the efficiency of the bioprocess in biosurfactant production is the critical micellar concentration (CMC). The CMC of the lipopeptide produced by *Salibacterium* sp. 4CTb was reported to be 15.1 mg/L [7], which is comparatively smaller than those described for other lipopeptides, ranging from 150–200 mg/L [31]. However, it is important to consider that these values may vary according to the composition of the medium and culture conditions. Interestingly, it is remarkable that the CMC of the lipopeptides described was different than the CMC reported in the synthetic surfactants. By way of example, the CMC of sodium dodecyl sulfate (SDS), tetradecyltrimethylammonium bromide (TTAB), cetylpyridinium chloride (CPC), and sodium bis(2-ethylhexyl) sulfosuccinate (AOT) are 2307, 1345, 350, and 1182 mg/L, respectively, which corroborates the outstanding performance of lipopeptides as surface-active agents [55,56]. Taken together, the above reports that the lower the CMC value the more significant the effect of the compound as a surfactant and the greater its industrial application potential [57].

### 3.2. Impact of k_L_a on Lipopeptide Performance

**Figure 3:**
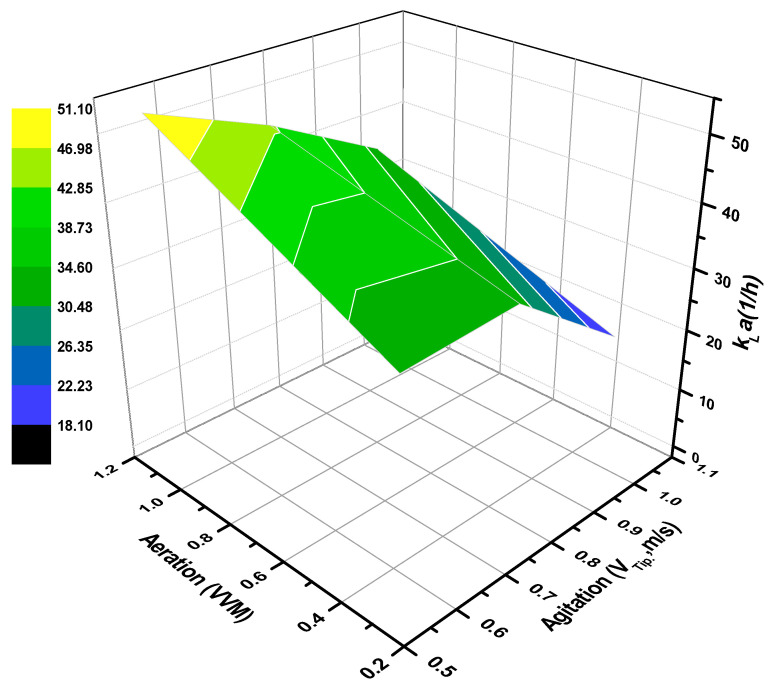
Response surface methodology of *k_L_a* behavior as a function of impeller tip velocity (m/s) and aeration (vvm).

Oxygen consumption in aerobic cultures has proven to be a key concern for bioprocess scale-up, thus, effective manipulation of the volumetric mass transfer coefficient (*k_L_a*) between laboratory and pilot scales could determine the main pathways to reaching an industrial scale [35]. As previously described, the variation in agitation and aeration rates significantly influenced *Salibacterium* sp. 4CTb to synthesize the lipopeptide. Nevertheless, for further scale-up purposes, the agitation rate is expressed as impeller tip speed [43,58], as expressed through Equation (7):

(7) $$V_{Tip}\left(\frac{m}{s}\right)=\left(\frac{N}{60}\right)*\left(\pi \right)*\left(D\right)$$

where *N* represents the agitation rate, and *D* is the Rushton impeller diameter. The different runs of cultures showed fluctuations in *k_L_a* that were influenced by several factors, such as the impeller tip speed, aeration, and the presence of biosurfactant [45]. According to Figure 3, it was observed that in the region near 1.2 vvm–0.57 m/s, it is possible to reach the maximum oxygen transfer rate (*k_L_a* = 33.1 1/h); however, by contrasting this region with the response surface in Figure 2, it is remarkable that E_24_% slightly tops 40%. On the other hand, the operating conditions of 0.48 vvm and 0.66 m/s, which yielded, for the most part, the lipopeptide production with E_24_% = 74.55%, experimentally generated an oxygen transfer rate of *k_L_a* = 31 1/h, quite proximal to the previously described. Measurements in the surface tension were not performed; and yet, the generation of tensoactive compounds is capable of favoring oxygen transfer in culture media [59]. Moreover, the recovery of the lipopeptide could be compromised if the culture is not harvested at an optimal growth stage. Taken together with the above observations, high rates of oxygen transfer to the bioreactor might not be favorable to lipopeptide synthesis [60], due to foam generation and the microorganism’s preference for biomass production.

## 4. Conclusions

The proposed design of experiments (DOE) proved to be statistically efficient for the improvement of lipopeptide production through *Salibacterium* sp. 4CTb in stirred-tank bioreactors. Moreover, it was corroborated that agitation and aeration are critical parameters of the bioprocess, where E_24_% reached up to 74.55% and the oxygen transfer rate (*k_L_a*) reached 31 1/h. It is clear that *Salibacterium* sp. 4CTb is a promising halophilic microorganism for larger-scale production of lipopeptide, therefore for future studies, it is critical to explore the *k_L_a* in various bioreactor volumes and to maintain fixed various process criteria, such as the oxygen transfer rate, bioreactor geometry, and culture medium, among others. On the other hand, it is interesting to consider that the formulation of the culture medium presented in this work represents a high cost, therefore, the pursuit of a culture medium from industrial waste is an innovative approach for future applications. Moreover, among the obstacles present within the scale-up, the high concentration of NaCl is a factor attributed to high foaming and could lead to gradients in the composition of the culture media and *Salibacterium* sp. 4CTb may not assimilate nutrients favorably.

The optimization of lipopeptide production lays the foundation for targeting various application areas, such as oil recovery, heterogeneous biocatalysis, and agriculture, among others, with the purpose of directing future research towards the substitution of chemical surfactants by demonstrating the potential of biological surfactants in all industrial sectors due to their environmental, monetary and handling advantages. Within this context, biosurfactants have shown low cytotoxicity, which makes them an important tool nowadays, especially during the COVID-19 pandemic, and therefore their application in surface sanitizers, drug distributors, and additives in face masks, among others, is critical. In light of the above, it is clear that biosurfactants possess a high versatility in these areas of application.

## Figures and Tables

**Table 1:** Coded and real variables of the 2*^k^* central composite design (CCD). Factors are aeration (vvm) and agitation (rpm). The factors levels were 0.5 vvm, 0.9 vvm, and 1.2 vvm for aeration, 500 rpm, 700 rpm, and 900 rpm for agitation. All runs were performed at 37 °C and pH 9.

Run	Coded Variables		Real Variables	
	X1	X2	Z1 (vvm)	Z2 (rpm)
1	−1	−1	0.5	500
2	−1	1	0.5	900
3	1	−1	1.2	500
4	1	1	1.2	900
5	0	0	0.9	700
6	0	0	0.9	700
7	0	0	0.9	700
8	−1.41	0	0.34	700
9	1.41	0	1.32	700
10	0	−1.41	0.9	980
11	0	1.41	0.9	420

The significance of the mathematical models was tested through analysis of variance (ANOVA) with a significance level of 0.05. All analyses were performed by the commercial package OriginPro 8.5 (Originlab Corporation, Northampton, MA, USA).

## Data Availability

Not applicable.
